# Eight Orthostatic Haemodynamic Patterns in The Irish Longitudinal Study on Ageing (TILDA): Stability and Clinical Associations after 4 Years

**DOI:** 10.3390/geriatrics6020050

**Published:** 2021-05-11

**Authors:** David Moloney, Silvin P. Knight, Louise Newman, Rose Anne Kenny, Roman Romero-Ortuno

**Affiliations:** 1 The Irish Longitudinal Study on Ageing (TILDA), Trinity College Dublin, D02 R590 Dublin, Ireland; silvin.knight@tcd.ie (S.P.K.); louise.newman@tcd.ie (L.N.); rkenny@tcd.ie (R.A.K.); romeroor@tcd.ie (R.R.-O.); 2 Falls and Syncope Unit, Mercer’s Institute for Successful Ageing, St James’s Hospital, D08 NHY1 Dublin, Ireland; 3 Global Brain Health Institute, Trinity College Dublin, D02 PN40 Dublin, Ireland

**Keywords:** orthostatic hypotension, falls, syncope, longitudinal, physiological monitoring

## Abstract

Previous research cross-sectionally characterised eight morphological systolic blood pressure (SBP) active stand (AS) patterns using a clinical clustering approach at Wave 1 (W1) of the Irish Longitudinal Study on Ageing. We explored the longitudinal stability and clinical associations of these groupings at Wave 3 (W3), four years later. Eight AS groups had their clinical characteristics and AS patterns at W3 compared to W1. We explored longitudinal associations (new cognitive decline, falls, syncope, disability, and mortality) using multivariate logistic regression models. In total, 2938 participants (60% of Wave 1 sample) had adequate AS data from both W1 and 3 for analysis. We found no longitudinal stability of the eight AS groups or their morphological patterns between the waves. A pattern of impaired stabilisation and late deficit seemed more preserved and was seen in association with new cognitive decline (OR 1.63, 95% CI: 1.12–2.36, *p* = 0.011). An increase in antihypertensive usage seemed associated with reduced immediate SBP drops, improved AS patterns, and reduced orthostatic intolerance (OI). In pure longitudinal groups, AS patterns were not preserved after 4 years. AS patterns are longitudinally dynamic, and improvements after 4 years are possible even in the presence of higher antihypertensive burden.

## 1. Introduction

Orthostatic Hypotension (OH) is traditionally defined as a reduction in systolic blood pressure (SBP) of at least 20 mmHg or diastolic blood pressure (DBP) of at least 10 mmHg within 3 min of standing [1]. This definition is based on intermittent blood pressure measurements via sphygmomanometer during a 3-min orthostatic blood pressure assessment.

The reproducibility of orthostatic haemodynamic responses has been inconsistent across studies [2]. For example, orthostatic blood pressure responses measured with sphygmomanometer may not be reproducible in patients with documented symptomatic OH, particularly if autonomic function is normal and measurements are taken in the afternoon [3]. There is evidence that orthostatic blood pressure responses can be highly variable during the day in hospital inpatients [4,5], with poor day-to-day reproducibility [6], and even seasonal variation [7]. Furthermore, OH features found on an active stand (AS) using non-invasive beat-to-beat technology such as the Finometer have only demonstrated a moderate reproducibility at 6–8 weeks follow up [8].

Whilst OH may be a physiological marker of transient normal [9] and abnormal health states of no long-term relevance (e.g., episode of dehydration, viral illness, transient drug effects) [10], the long-term persistence of OH may be relevant to the development of serious systemic disorders that usually take years to manifest clinically. For example, OH has been associated with the development of cognitive decline [11] and dementia [12]. Therefore, the long-term study of orthostatic hemodynamic patterns may be relevant for the early identification of the latter conditions, under the hypothesis that at-risk patients may be more likely to manifest the long-term persistence of an abnormal orthostatic hemodynamic pattern. These individuals may also be more likely to experience new falls and/or syncope, accelerated disability, and increased mortality.

Our previous research cross-sectionally characterised eight morphological SBP AS patterns based on a clinical clustering approach at Wave 1 (W1) of TILDA [13]. Our hypothesis was that there would be poor longitudinal persistence of the eight groupings due to the multifactorial nature of normal haemodynamic responses. However, long-term persistence of a particular AS pattern could be a sign of underlying progressive disease or physiological dysfunction with reduced compensatory ability; in other words, a preserved haemodynamic pattern might be a sign that the body is not capable of the same degree of variability in haemodynamic response to standing compared to healthy individuals.

To address the above hypothesis, we set out to explore the longitudinal stability and clinical associations of these groupings at Wave 3 (W3), which was approximately four years later.

## 2. Methods

### 2.1. Sample

Analysis was conducted on data from the health assessments of TILDA W1 (June 2009–June 2011) and W3 (June 2014–December 2015). TILDA participants were selected using multi-stage, stratified random sampling that identified 640 geographical areas, stratified by socio-economic characteristics, and selected 40 households within each area [14,15]. The Irish GeoDirectory listing of all residential addresses provided the sampling frame. Further details of the study design, sampling and methodology have been described elsewhere [16,17,18]. Participants with missing or inadequate AS data were not included.

### 2.2. Active Stand Protocol

Participants underwent an AS test with the Finometer MIDI device (Finapres Medical Systems BV, Amsterdam, The Netherlands), performed by trained research nurses and recorded at 200 Hz. Participants underwent the AS following approximately 10 min of supine rest. Baseline BP was calculated as the mean value between 60 and 30 s before standing. Data was downsampled to 1 Hz. Two smoothing filters were applied, a 10-point moving average filter and an 11-point median filter. Onset of the stand was detected via an algorithm using data from the Finometer height correction unit [19]. Here we utilised BP response data up to 120 s post-stand, at 10-s intervals.

### 2.3. Longitudinal Variables

New cognitive decline was defined as a decrease in Mini-Mental State Examination (MMSE) score from W1 to W3 that was ≥2. On average, a 1–3 point decrease in MMSE is indicative of a meaningful decline [20].

New falls and new syncope were defined as not reporting any recent falls/syncope at W1 and subsequently reporting recent falls/syncope at W3. Recent falls/syncope were defined as having had a fall/syncope in the past 12 months.

New basic activities of daily living (ADL) disability and independent activities of daily living (IADL) disability were defined as reporting ≥1 new ADL/IADL disability at W3 when compared to W1. The six ADL questions were: difficulties with dressing, walking across a room, bathing or showering, eating, getting in or out of bed, and using the toilet. The six IADL questions were: difficulties with preparing a hot meal, doing household chores, shopping for groceries, making telephone calls, taking medications, and managing money.

Mortality data was obtained by linking survey respondents to their death certificate information held centrally by the General Register Office, where every death in the Republic of Ireland must be registered [21].

### 2.4. Clinical Characterisation Variables

The following variables were used to compare the eight groups between W1 and W3 and also to compare those with complete and missing data at W3: age (years), sex, a binary Fried’s frailty phenotype category (non-frail vs. pre-frail/frail) [22], time taken to stand during the AS [19], multimorbidity (history of two or more self-reported diseases among the following: myocardial infarction, heart failure, angina, atrial fibrillation, hypertension, hypercholesterolemia, stroke, diabetes mellitus, chronic obstructive pulmonary disease, asthma, arthritis, osteoporosis, cancer, Parkinson’s disease, and hip fracture). We also characterised the groups according to usage of antihypertensive and psychotropic medications. Polypharmacy was defined as concomitant use of five or more regular medications. Orthostatic intolerance (OI) was defined as present if a participant felt dizzy, light-headed or unsteady during the AS. Healthcare utilisation was assessed using general practitioner (GP) visits in the past year, which was a self-reported variable.

### 2.5. Statistical Analyses

Statistical analyses were performed in Stata version 14.1 (StataCorp, College Station, TX, USA). In order to study pure longitudinal samples, we selected within each of the original baseline AS groupings [13], those participants who also had AS data at W3. Descriptive statistics were given as mean with standard deviation (SD), median with interquartile range (IQR), or number (*n*) with percentage (%). Differences in the clinical characteristics between those with complete and missing data at W3 were assessed using the Mann–Whitney U test for continuous variables and the Chi-squared test for categorical (e.g., dichotomous) variables.

Graphical visualisations comparing the W1 and W3 eight AS groups were generated; presented as change in SBP from baseline during the active stand at each 10 s timepoint, with +/− 95% confidence intervals also shown.

For the longitudinal associations between the AS groups and the outcome variables (new cognitive decline, new falls, new syncope, new ADL disability, new IADL disability, mortality), three logistic regression models were fitted for each outcome: Model A, a univariate model with the AS group as independent variable using the no deficits group as reference; model B, a multivariate model controlling for the fixed effects of age and sex and model C, a multivariate model controlling for the fixed effects of age, sex, baseline SBP, time to stand, Fried’s frailty status, baseline MMSE, multimorbidity, polypharmacy, and use of antihypertensive, antidepressant, benzodiazepine, and Z-drug medications.

In these models, the threshold for statistical significance was set at *p* < 0.05.

### 2.6. Ethics

Ethical approval for each wave of TILDA was obtained from the Faculty of Health Sciences Research Ethics Committee in Trinity College Dublin. Participants were provided with sufficient information to make an informed decision about their participation including sufficient advance notice of the study. Written consent was obtained for separate components of the study (e.g., interview, health assessment); participants were able to refuse to take part in or withdraw at any time without providing justification. TILDA data collection involves minimal risk, invasion, burden and discomfort to participants, though there are potential ethical issues that may arise, e.g., excessive time commitment or distress due to the nature of some questions being asked, or immediate and/or unforeseen medical concerns, which are all addressed as per the standard operating procedures and terms of the ethical approvals [17].

## 3. Results

In total, 8174 participants over the age of 50 were recruited to W1 of the TILDA study, of whom 5034 attended the health assessment centre. There were 4905 participants with adequate AS data for analysis, of whom 4899 had complete data for the generation of the eight AS groups [13]. A second health assessment, W3 of the TILDA study, was performed approximately four years later. There were 2938 participants (60% of the W1 sample) who had adequate AS data from both W1 and W3 for analysis. See Figure S1 for a flow diagram of the study attrition.

The attrition rate was not consistent across all groups: Group 2 had the largest attrition with 37.2% of W1 sample available for analysis; and Groups 3 (63.2% of W1 sample) and 7 (64.1% of W1 sample) had the smallest attrition. The other group’s attrition rates were as follows: Group 1 (53.7% of W1 sample), Group 4 (57.9% of W1 sample), Group 5 (45.5% of W1 sample), Group 6 (47.4% of W1 sample), and Group 8 (61.4% of W1 sample).

### 3.1. Morphological Pattern Stability, Clinical Characteristics, and Longitudinal Associations

**Figure 1:**
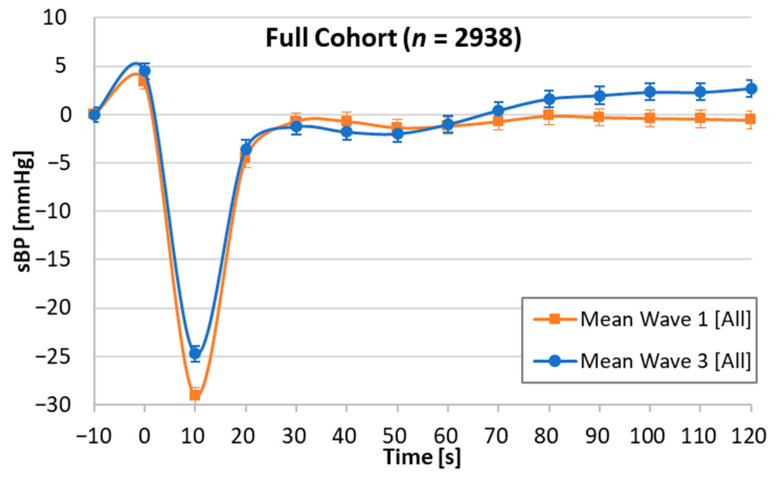
Graphical visualisation of the orthostatic systolic blood pressure active stand profiles of the full cohort at Wave 1 and Wave 3. SBP: systolic blood pressure.

Figure 1 shows the W1 and W3 morphological AS pattern superimposed in the full cohort (*n* = 2938). It appears that at W3, individuals had a lesser SBP drop at 10 s post-stand and an overshoot above SBP baseline from 90 s post-stand. When comparing W1 and W3 (Table 1), the prevalence of self-reported hypertension decreased (37.2 vs. 34.5%); however, there was an increase in mean baseline SBP (134.9 vs. 140.7 mmHg) and prescribed antihypertensives (27.3 vs. 36.3%).

**Figure 2:**
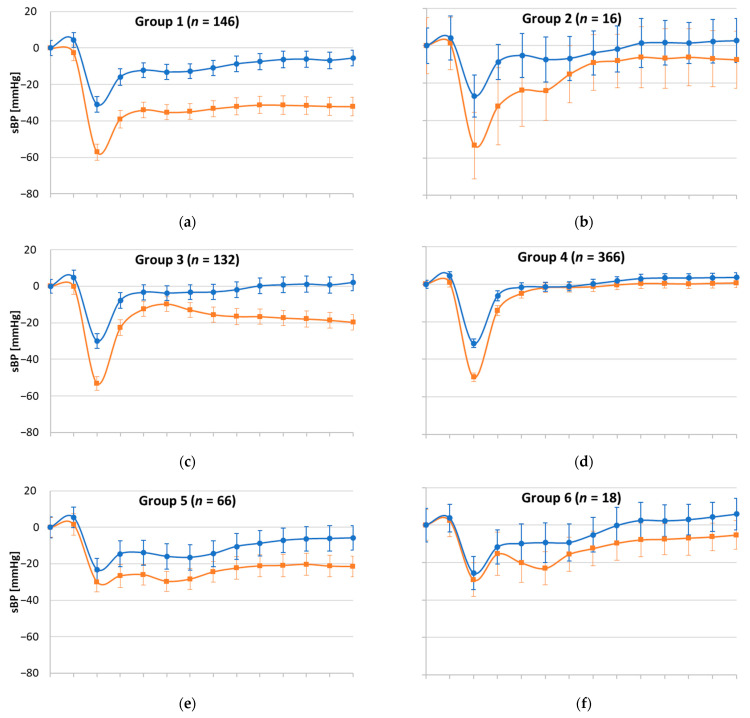

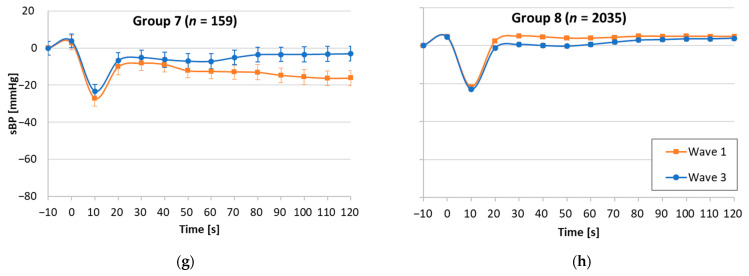
Graphical visualisation of the orthostatic systolic blood pressure active stand profiles of the eight groups at Wave 1 and Wave 3. SBP: systolic blood pressure. (**a**): Group 1. (**b**): Group 2. (**c**): Group 3. (**d**): Group 4. (**e**): Group 5. (**f**): Group 6. (**g**): Group 7. (**h**): Group 8.

Figure 2 shows W1 and W3 patterns for the eight AS groups. The 146 participants who at W1 had displayed a full-deficit pattern (i.e., immediate, stabilisation and late deficits) seemed to have a much-improved pattern at W3 throughout the stand. As Table 1 shows, only 26%, 35%, and 43% of Group 1 participants had these three deficits at W3, respectively. There was an 11.7% reduction in OI symptoms during the AS. This is despite a 9.6% increase in multimorbidity over 4 years, including a 2.7% in diabetes and an 11.6% increase in prescribed antihypertensives. As Table 2 shows, this group had no longitudinal associations with any of the outcomes studied.

Groups 2 and 6 were very small (*n* = 16 and 18, respectively) and there was little statistical power to detect statistically significant differences in AS morphology.

The 132 participants who at W1 had displayed a pattern of immediate and late deficits (Group 3) also seemed to have an improved longitudinal pattern, with only 23.5% and 21.2% of participants having those deficits at W3. In this group, the prevalence of self-reported hypertension reduced by 15.9%, with a 14.4% increase in antihypertensives and an 18.2% reduction in OI. Again, in this group there were no significant longitudinal associations with negative clinical outcomes.

In Group 4 (*n* = 366), the only morphological difference visually seemed to be an improvement in the initial drop by W3 (26.5% versus 100% at W1), with similar recoverability. The prevalence of self-reported hypertension reduced by 5.8% with a 38.0% increase in antihypertensives and there was an 11.5% reduction in OI. There was a 5.8% increase in frailty status and there was a statistically significant longitudinal association with new falls: OR 1.36, 95% CI: 1.07–1.72, *p* = 0.012 (Table 2).

Group 5 (*n* = 66), originally characterised by stabilisation and late deficits, seemed to have a more preserved longitudinal morphological pattern (Figure 2) with a suggestion of a better recovery (37.9% and 45.5% recurrence of deficits, respectively). As regards clinical characteristics, there was a 22.7% increase in frailty. There was a statistically significant longitudinal association with new cognitive decline: OR 1.63, 95% CI: 1.12–2.36, *p* = 0.011 (Table 2).

In Group 7 (*n* = 159), originally characterised by late deficit only, there was only 32.1% of that deficit by W3. The prevalence of self-reported hypertension reduced by 9.5% with a 17.0% increase in antihypertensives and there was an 15.7% reduction in OI. There were two new cases of Parkinson’s disease in this group. There was a statistically significant longitudinal association with new falls: OR 1.47, 95% CI: 1.03–2.09, *p* = 0.034 (Table 2).

The large Group 8 (*n* = 2035), characterised by no baseline deficits, seemed to have lost some of the post-stand overshoot response during the first minute post-stand (Figure 2). By W3, 10.8% had immediate, 7.2% stabilisation, and 12.7% late deficits (Table 1). There was a 10.5% increase in frailty, a 7.5% increase in antihypertensives and an 8.7% reduction in OI.

There were no differences in GP visits in the past year between the eight groups.

In terms of the independent effects of the controlling variables in the regression mod-els where AS groups were independently significant (new falls and new cognitive decline), the following effects were found: for new falls, independently significant effects were being on benzodiazepines (OR 1.79, 95% CI: 1.17–2.73, *p* = 0.007), multimorbidity (OR 1.27, 95% CI: 1.05–1.54, *p* = 0.014), female sex (OR 1.32, 95% CI: 1.11–1.57, *p* = 0.002) and age (OR 1.03, 95% CI: 1.02–1.04, *p* < 0.001). In terms of new cognitive decline, independent predictors were also frailty (OR 1.27, 95% CI: 1.08–1.48, *p* = 0.003), female sex (in a protective fashion: OR 0.86, 95% CI: 0.74–0.99, *p* = 0.033), and age (OR 1.02, 95% CI: 1.01–1.03, *p* < 0.001).

### 3.2. Attrition Characteristics

When comparing those who attended their second health assessment at W3 with those who did not, the group who were missing at W3 had, at baseline, a more adverse health profile, including being older, more multimorbid (42.2 vs. 50.8%), more prefrail/frail (23.4 vs. 33.6%), hypertensive (37.2 vs. 43.4%), more likely to have polypharmacy (13.5 vs. 22.3%), and be taking psychotropic medications (7.5 vs. 11.5%) (Table 3).

## 4. Discussion

In this large population-based study of Irish participants aged 50+ undergoing continuous orthostatic BP measurements, we explored the longitudinal associations and the stability of both morphological and clinical features of eight clinically clustered groups.

After four years, we observed common patterns of ageing across all groups with increasing levels of polypharmacy [23] and multimorbidity [24]; overall, our results suggest a healthier response in W3 compared to the same W1 participants. However, adverse health profiles were overrepresented in those who did not have a repeat AS at W3.

When comparing the overall SBP changes from baseline between W1 and W3, the major difference was that the depth of the initial drop seemed deeper at W1 than at W3. Recently, the Malaysian Elders Longitudinal Research (MELoR) study found that people with an immediate drop had better physical performance and were less likely to be frail [25].

Consistent with the MELoR study, we found a lower prevalence of immediate drop at W3 and participants at W3 were older and had higher rates of prefrailty/frailty. However, there seemed to be a decrease in mean time to stand (7.6 vs. 7.2 s) when comparing W1 to W3, which is not consistent with the theory that a faster standing speed leads to a greater immediate drop. The faster standing speed may have resulted in an earlier drop that recovered by the 10-s mark and we may not have captured it.

We found no longitudinal stability of the eight AS patterns four years later and this study echoes similar findings found by Finucane et al. with the Irish SHARE cohort [8]. This may be due to the fact that the reproducibility of an AS can be influenced by many factors including the underlying cause of OH [3], the time of year [7], most recent meal [26], hydration status [27], and medication usage [28].

Our main finding was that Group 5, originally characterised by stabilisation and late deficits, seemed to have a preserved longitudinal morphological pattern and a statistically significant association with new cognitive decline. It is possible that this morphological pattern, when persisting over time, may be a marker of cognitive decline. OH measured using intermittent blood pressure measurements has been associated with incident dementia in longitudinal studies such as the Malmö Preventive Project [29] and the Three-City Study Cohort [30]. It is hypothesised that OH can lead to periods of cerebral hypoperfusion, resulting in end organ damage and therefore putting patients at a higher risk of developing cognitive decline/dementia.

The immediate drop has been associated cross-sectionally with falls in community-dwelling older adults [31] and it is thought that the healthier the person is, the faster they can stand up [19]. There is no obvious haemodynamic reason as to why Groups 4 and 7 would be associated with developing new falls four years later, but we noted independently significant effects of benzodiazepines, multimorbidity, female sex, and age, all of which are associated with increased falls risk. Another possible factor could be the significant increases in antihypertensive medications in these groups (38% in Group 4 and 17% in Group 7). Falls are common in older people, with 30% of community dwelling older adults falling each year [32], and are due to an interaction of multiple risk factors, both intrinsic and extrinsic [33].

Both the Antihypertensive and Lipid-Lowering Treatment to Prevent Heart Attack Trial (ALLHAT) [34] and the Fall-Risk-Increasing Drugs (FRID) studies reported an inconsistent relationship between antihypertensive medications and falls, with no relationship found between most antihypertensive regimes and falls. ALLHAT found that amlodipine was associated with an increased risk of falls in the first year of the prescription and FRID found that loop diuretics were associated with increased falls.

The increase in antihypertensive prescriptions at W3 can be mainly attributed to the increase in prescriptions for angiotensin-converting enzyme inhibitors and angiotensin receptor blockers (ACEI/ARBs) (18.3 vs. 25.4%) and calcium-channel blockers (CCBs) (6.8 vs. 10.0%). Every group except for Group 2 had a large increase in ACEI/ARB usage and Groups 2, 3, and 4 had increases in CCB usage.

The FRID study found that beta-blockers are associated with a reduced risk of falls; however, they noted that more recent studies only demonstrated a positive association between non-selective beta-blockers and falls [35]. This contrasts with our study, which found that the biggest increases in beta-blocker prescription were seen in Groups 4 (4.1 vs. 12.1%) and 7 (8.9 vs. 14.5%), which were the groups associated with developing future falls at W3. Furthermore, in our study, non-selective beta-blockers only accounted for 5.1% of the total beta-blocker prescriptions and they were evenly spread across all groups.

Another important finding of our study is that an increase in antihypertensive usage was seen, in many cases, with improved morphological patterns and reduced proportions of OI. This is consistent with the findings of a recent systematic review and meta-analysis by Juraschek et al. that found that treating hypertension improves orthostatic BP patterns and reduces OH [36]. It is hypothesised that this may be secondary to the improved baroreflex sensitivity [37,38] and cerebral blood flow [39] associated with antihypertensive therapy. One limitation of the meta-analysis is that OH was measured using intermittent blood pressure measurements via sphygmomanometer, which typically misses the first minute of a person’s haemodynamic response.

In terms of new cognitive decline, as well as AS Group 5 membership, independent predictors were also frailty, female sex in a protective fashion, and age. The identification of frailty in this model is interesting because physical activity interventions have been shown to delay and reverse frailty in populations [40] and improve OI [41], as well as potentially having neuroprotective effects [42]. Therefore, the study as to how exercise may protect cognition by improving orthostatic hemodynamics merits further investigation outside our observational epidemiological design.

### Limitations

There was a rate of attrition of 40% of participants between W1 and W3 and we can see, from comparing the baseline characteristics of those who had an AS at W3 vs. those who did not, that there is a ‘healthy returner effect’. The participants who did not have an AS at W3 were more multimorbid and more prefrail/frail at W1. Due to attrition, some groups had small numbers, especially Group 2 (*n* = 18) and Group 6 (*n* = 18), which means that meaningful conclusions cannot be made on these groups.

In addition, potential clinical limitations are that the time between W1 and W3 was quite short (four years) for the development of infrequent longitudinal adverse conditions that develop over a long time, and there were low rates of new diagnoses of diabetes or Parkinson’s disease.

Between the two waves, there was a decrease in self-reported hypertension but an increase in baseline SBP measurements and antihypertensive usage. This may be explained by the fact that participants believed that their hypertension was ‘treated’ by the antihypertensives they were prescribed so did not report self-reported hypertension. As regards this and other self-reported clinical outcomes, our findings are limited by recall bias.

In view of physical frailty being independently implicated in the prediction of new cognitive decline, a limitation is that other than self-reported activity levels included in the frailty definition, we did not have more objective measures of physical activity levels to include as an independent variable to the regression models.

Another limitation is that we did not consider the potential contribution of heart rate to the observed blood pressure responses across the different groups. Heart rate data was not included in the results because in clinical practice, only blood pressure is considered in the definition of OH. The observation of the orthostatic HR response can provide additional information as to how hydration status/medications/autonomic function may be contributing to the blood pressure patterns, but a difficulty is that there are no guidelines or consensus on HR cut-offs for the interpretation of orthostatic HR responses.

## 5. Conclusions

In conclusion, our study found that there is no longitudinal stability of the eight AS groups four years later and that there was a decrease in the prevalence of an immediate orthostatic drop as people aged. The poor persistence of AS patterns indicates that, while the AS is a useful cross-sectional investigation of a person’s haemodynamic response to standing, it becomes less useful to predict future AS patterns.

This study highlights the potential longitudinal association between prolonged periods of cerebral hypoperfusion and the development of cognitive decline and highlights the need to identify and treat the presence of OH to help preserve future cognitive function.

Finally, an increase in antihypertensive usage was associated with reduced immediate SBP drops, improved AS patterns, and reduced OI. This has clinical implications for antihypertensive prescribing in community-dwelling older adults as increasing rates of antihypertensive usage appears to improve SBP responses and symptoms.

## Figures and Tables

**Table 1:** Characterisation of the overall sample and the eight active stand groups at Wave 1 and Wave 3.

	W1	W3	W1	W3	W1	W3	W1	W3	W1	W3	W1	W3	W1	W3	W1	W3	W1	W3
	Entire Cohort (*n* = 2938)		Group 1 (*n* = 146)		Group 2 (*n* = 16)		Group 3 (*n* = 132)		Group 4 (*n* = 366)		Group 5 (*n* = 66)		Group 6 (*n* = 18)		Group 7 (*n* = 159)		Group 8 (*n* = 2035)	
**Mean Baseline SBP** (SD) [mmHg]	134.9 (21.9)	140.7 (24.4)	148.8 (25.5)	146.9 (25.0)	131.0 (30.8)	141.7 (19.4)	143.6 (22.6)	141.3 (21.9)	139.6 (22.3)	143.6 (20.1)	142.1 (22.1)	141.9 (23.8)	147.4 (17.9)	154.5 (19.5)	145.2 (24.7)	146.6 (23.8)	131.4 (20.1)	139.1 (20.8)
**% immediate deficit**	23.6	14.4	100	26.0	100.0	12.5	100.0	23.5	100.0	26.5	0	12.1	0	22.2	0	13.8	0	10.8
**% stabilisation deficit**	10.2	10.5	100	34.9	100	12.5	0	15.2	0	10.4	100	37.9	100	22.2	0	13.8	0	7.2
**% late deficit**	17.8	17.0	100	43.2	0	18.8	100	21.2	0	17.5	100	45.5	0	16.7	100	32.1	0	12.7
**Mean time to stand in seconds** (SD)	7.6 (3.0)	7.2 (2.9)	7.9 (2.7)	7.4 (3.2)	8.3 (3.5)	7.1 (2.4)	7.3 (2.3)	7.2 (2.6)	7.5 (2.8)	7.1 (2.7)	9.7 (4.5)	8.3 (3.6)	9.0 (3.8)	8.9 (5.7)	8.1 (3.2)	7.5 (3.4)	7.5 (2.9)	7.2 (2.9)
**Mean age in years** (SD)	59.8 (7.9)	64.2 (7.9)	62.8 (9.1)	67.1 (9.1)	65.4 (9.6)	69.9 (9.7)	59.6 (7.2)	64.0 (7.2)	60.5 (7.7)	65.0 (7.6)	64.9 (7.9)	69.2 (7.9)	64.9 (9.4)	69.2 (9.6)	60.1 (8.1)	64.5 (8.1)	59.2 (7.7)	63.5 (7.7)
**Sex** (*n* (%)) [female]	1609 (54.8)	1609 (54.8)	93 (63.7)	93 (63.7)	8 (50.0)	8 (50.0)	93 (70.4)	93 (70.4)	211 (57.7)	211 (57.7)	42 (63.6)	42 (63.6)	9 (50.0)	9 (50.0)	100 (62.9)	100 (62.9)	1053 (51.7)	1053 (51.7)
**Multimorbidity** (*n* (%))	1239 (42.2)	1302 (44.4)	66 (45.2)	80 (54.8)	9 (56.3)	11 (68.8)	52 (39.4)	62 (47.0)	164 (44.8)	173 (47.4)	34 (51.5)	38 (57.6)	11 (61.1)	9 (50.0)	70 (44.0)	74 (46.5)	833 (40.9)	855 (42.1)
**No. of chronic conditions** (mean (SD))	1.4 (1.1)	1.5 (1.2)	1.4 (1.1)	1.8 (1.5)	1.9 (1.2)	2.0 (1.2)	1.3 (1.1)	1.5 (1.3)	1.4 (1.0)	1.6 (1.3)	1.6 (1.2)	1.9 (1.3)	1.8 (1.1)	1.6 (1.0)	1.4 (1.1)	1.6 (1.2)	1.3 (1.1)	1.4 (1.2)
**No. of cardiovascular conditions** (mean (SD))	0.1 (0.5)	0.1 (0.3)	0.2 (0.5)	0.2 (0.4)	0(0)	0.2 (0.5)	0.1 (0.4)	0.1 (0.4)	0.2 (0.5)	0.1 (0.4)	0.1 (0.4)	0.1 (0.4)	0.1 (0.3)	0.1 (0.3)	0.1 (0.5)	0.1 (0.3)	0.1 (0.4)	0.1 (0.3)
**Hypertension** (*n* (%))	1090 (37.2)	1013 (34.5)	60 (41.1)	57 (39.0)	6 (37.5)	7 (43.8)	60 (45.5)	39 (29.6)	169 (46.6)	149 (40.8)	27 (40.9)	28 (42.4)	8 (44.4)	9 (50.0)	75 (47.2)	60 (37.7)	685 (33.7)	664 (32.7)
**Diabetes** (*n* (%))	147 (5.0)	190 (6.5)	10 (6.9)	14 (9.6)	1 (6.3)	1 (6.3)	5 (3.8)	9 (6.8)	16 (4.4)	17 (4.6)	3 (4.6)	3 (4.6)	2 (11.1)	3 (16.7)	6 (3.8)	7 (4.4)	104 (5.1)	136 (6.7)
**Parkinson’s** (*n* (%))	6 (0.2)	12 (0.4)	0 (0)	1 (0.7)	0 (0)	0 (0)	0 (0)	0 (0)	0 (0)	0 (0)	1 (1.5)	1 (1.5)	0 (0)	0 (0)	2 (1.3)	4 (2.5)	3 (0.2)	6 (0.3)
**Binary Frailty (prefrail/frail)** (*n* (%))	676 (23.4)	799 (32.4)	44 (31.0)	39 (32.5)	5 (31.3)	4 (30.8)	32 (24.2)	35 (32.7)	82 (22.8)	89 (28.6)	18 (27.3)	24 (50.0)	3 (16.7)	3 (20.0)	49 (31.8)	45 (33.1)	443 (22.1)	560 (32.6)
**Antihypertensives** (*n* (%))	803 (27.3)	1066 (36.3)	50 (34.3)	67 (45.9)	6 (37.5)	6 (37.5)	32 (24.2)	51 (38.6)	105 (28.7)	149 (40.7)	27 (40.9)	30 (45.5)	6 (33.3)	8 (44.4)	39 (24.5)	66 (41.5)	538 (26.4)	689 (33.9)
**ACEI/ARB** (*n* (%))	536 (18.3)	747 (25.4)	27 (18.6)	39 (26.7)	4 (25.0)	4 (25.0)	18 (13.6)	37 (28.0)	76 (20.8)	105 (28.7)	17 (26.2)	25 (37.9)	2 (11.8)	4 (22.2)	27 (17.1)	51 (32.1)	365 (18.0)	482 (23.7)
**Beta-blockers** (*n* (%))	274 (9.4)	369 (12.6)	22 (15.2)	28 (19.2)	0 (0)	0 (0)	12 (9.1)	14 (10.6)	38 (10.4)	60 (16.4)	12 (18.5)	11 (16.7)	1 (5.9)	2 (11.1)	14 (8.9)	23 (14.5)	175 (8.6)	231 (11.4)
**Diuretics** (*n* (%))	127 (4.3)	127 (4.3)	9 (6.2)	12 (8.2)	1 (6.3)	2 (12.5)	5 (3.8)	3 (2.3)	11 (3.0)	16 (4.4)	5 (7.7)	6 (9.1)	1 (5.9)	0 (0)	8 (5.1)	8 (5.1)	87 (4.3)	80 (3.9)
**CCBs** (*n* (%))	200 (6.8)	293 (10.0)	13 (9.0)	20 (13.7)	3 (18.8)	4 (25.0)	5 (3.8)	16 (12.1)	15 (4.1)	44 (12.0)	7 (10.8)	9 (13.6)	4 (23.5)	3 (16.7)	9 (5.7)	13 (8.2)	144 (7.1)	184 (9.0)
**Alpha blockers** (*n* (%))	28 (1.0)	30 (1.0)	4 (2.8)	3 (2.1)	0 (0)	0 (0)	0 (0)	0 (0)	3 (0.8)	2 (0.6)	1 (1.5)	1 (1.5)	0 (0)	0 (0)	2 (1.3)	1 (0.6)	18 (0.9)	23 (1.1)
**Psychotropics** (*n* (%))	219 (7.5)	300 (10.2)	20 (13.7)	24 (16.4)	2 (12.5)	3 (18.8)	8 (6.1)	9 (6.8)	36 (9.8)	46 (12.6)	6 (9.1)	10 (15.2)	0 (0)	2 (11.1)	17 (10.7)	20 (12.6)	130 (6.4)	186 (9.1)
**Orthostatic intolerance** (*n* (%))	1150 (39.2)	853 (29.1)	68 (46.6)	51 (34.9)	8 (50.0)	5 (31.3)	57 (43.2)	33 (25.0)	168 (45.9)	126 (34.4)	23 (34.9)	19 (28.8)	7 (38.9)	5 (27.8)	73 (45.9)	48 (30.2)	746 (36.7)	566 (28.0)
**GP visits in past year** (mean (SD))	3.1 (4.4)	3.2 (3.1)	3.5 (3.5)	3.5 (3.2)	3.3 (3.1)	3.0 (2.1)	3.1 (3.3)	3.1 (2.4)	2.9 (3.3)	3.3 (3.0)	3.4 (3.8)	3.3 (3.2)	3.6 (2.3)	3.0 (2.4)	3.6 (3.6)	3.7 (2.9)	3.0 (4.8)	3.1 (3.1)
**GP visits in past year** (median (IQR))	2 (3)	2 (3)	2.5 (3)	3 (3)	2 (2.5)	2 (3.5)	2 (3)	2 (2)	2 (3)	2 (3)	2.5 (3)	2 (2)	2.5 (3)	3 (3)	3 (3)	3 (3)	2 (3)	2 (3)

Abbreviations: SD: standard deviation; SBP: systolic blood pressure; ACEI: angiotensin-converting-enzyme inhibitors; ARB: angiotensin receptor blockers; CCB: calcium channel blockers; GP: general practitioner.

**Table 2:** Results of the fully adjusted logistic regression models (Models C). Statistically significant results are highlighted in bold.

	Group 1		Group 2		Group 3			Group 4		Group 5		Group 6		Group 7	Group 8
	OR (95% CI)	*p*	OR (95% CI)	*p*	OR (95% CI)	*p*	OR (95% CI)	*p*	OR (95% CI)	*p*	OR (95% CI)	*p*	OR (95% CI)	*p*	OR (95% CI)
**New fall**	0.99 (0.68–1.42)	0.936	1.63 (0.76–3.48)	0.210	0.98 (0.64–1.50)	0.942	**1.36 (1.07–1.72)**	**0.012**	1.25 (0.79–1.96)	0.339	0.87 (0.33–2.30)	0.785	**1.47 (1.03–2.09)**	**0.034**	(Base)
**New faint**	0.68 (0.29–1.61)	0.380	1.58 (0.37–6.80)	0.538	0.94 (0.37–2.38)	0.902	0.85 (0.47–1.52)	0.578	0.83 (0.29–2.35)	0.722	0.89 (0.12–6.74)	0.910	0.28 (0.07–1.14)	0.076	(Base)
**New ADL**	1.04 (0.74–1.47)	0.822	1.58 (0.76–3.31)	0.223	0.94 (0.62–1.43)	0.775	0.92 (0.72–1.19)	0.531	1.44 (0.95–2.21)	0.089	1.08 (0.45–2.56)	0.866	0.87 (0.59–1.28)	0.480	(Base)
**New IADL**	1.01 (0.71–1.42)	0.975	1.54 (0.73–3.24)	0.252	0.97 (0.64–1.47)	0.890	0.84 (0.65–1.09)	0.181	1.39 (0.91–2.12)	0.129	0.87 (0.35–2.18)	0.767	0.83 (0.56–1.22)	0.336	(Base)
**New cognitive decline**	1.25 (0.93–1.67)	0.134	1.25 (0.63–2.50)	0.524	0.79 (0.54–1.14)	0.210	0.98 (0.79–1.22)	0.876	1.63 (1.12–2.36)	0.011	0.99 (0.46–2.16)	0.988	0.82 (0.58–1.15)	0.240	(Base)
**Mortality**	1.17 (0.60–2.29)	0.653	0.49 (0.06–3.80)	0.495	0.98 (0.48–2.76)	0.970	0.95 (0.53–1.69)	0.856	1.64 (0.77–3.49)	0.203	0.47 (0.06–3.81)	0.477	0.63 (0.22–1.76)	0.377	(Base)

Abbreviations: OR: odds ratio; CI: confidence interval; ADL: activities of daily living; IADL: instrumental activities of daily living.

**Table 3:** Comparison of Wave 1 baseline characteristics of both the overall sample and attrition sample.

Baseline Characteristic	Wave 3 Data Present	Wave 3 Data Missing	*p*	
	(*n* = 2938)	(*n* = 1962)		
**Mean age in years** (SD)	59.8 (7.9)	62.88 (9.7)	<0.001	MWU
**Female** (*n* (%))	1609 (54.8)	1094 (55.8)	0.481	chi
**Non-frail** (*n* (%))	2213 (76.6)	1258 (66.4)	<0.001	chi
**Pre-frail/Frail** (*n* (%))	676 (23.4)	637 (33.6)	<0.001	chi
**Mean time to stand** (SD)	7.34 (2.6)	8.07 (3.4)	<0.001	MWU
**Median MMSE** (IQR)	29 (2)	29 (2)	<0.001	MWU
**Multimorbidity** (*n* (%))	1239 (42.2)	997 (50.8)	<0.001	chi
**Atrial Fibrillation** (*n* (%))	52 (1.8)	57 (3.0)	0.008	chi
**Parkinson’s disease** (*n* (%))	6 (0.2)	9 (0.5)	0.114	chi
**Diabetes Mellitus** (*n* (%))	147 (5.0)	153 (7.8)	<0.001	chi
**Hypertension** (*n* (%))	1090 (37.2)	844 (43.4)	<0.001	chi
**Polypharmacy** (*n* (%))	396 (13.5)	434 (22.3)	<0.001	chi
**Antihypertensive**				chi
**Overall** (*n* (%))	803 (27.3)	750 (38.3)	<0.001	chi
**Beta blockers** (*n* (%))	274 (9.3)	289 (14.8)	<0.001	chi
**Diuretics** (*n* (%))	127 (4.3)	162 (8.3)	<0.001	chi
**ACE inhibitors/Angiotensin receptor blockers** (*n* (%))	536 (18.3)	511 (26.2)	<0.001	chi
**Calcium channel blockers** (*n* (%))	200 (6.8)	202 (10.4)	<0.001	chi
**Alpha blockers** (*n* (%))	28 (1.0)	43 (2.2)	<0.001	chi
**Psychoactive medications**				
**Overall** (*n* (%))	219 (7.5)	225 (11.5)	<0.001	chi
**Z-drugs** (*n* (%))	51 (1.7)	58 (3.0)	0.004	chi
**Benzodiazepines** (*n* (%))	63 (2.2)	77 (4.0)	<0.001	chi
**Antidepressants** (*n* (%))	146 (5.0)	135 (6.9)	0.005	chi
**Orthostatic Intolerance during active stand** (*n* (%))	1150 (39.2)	730 (37.3)	0.187	chi
**At least 1 fall in the past 12 months** (*n* (%))	564 (19.2)	396 (20.2)	0.395	chi
**At least 1 blackout in the past 12 months** (*n* (%))	129 (4.4)	97 (5.0)	0.369	chi
**Lifetime history of syncope** (*n* (%))	589 (20.1)	374 (19.1)	0.398	chi
**Mean baseline SBP** (SD) [mmHg]	134.94 (21.9)	137.04 (22.8)	<0.001	MWU
**Mean baseline HR** (SD) [bpm]	64.52 (9.6)	65.61 (10.3)	<0.001	MWU

Abbreviations: MWU: Mann–Whitney U test (unpaired data); chi: Chi-square test; SD: standard deviation; IQR: interquartile range; bpm: beats per minute.

## Data Availability

TILDA provides access to the datasets for research use through anonymised publicly accessible dataset files, and through an on-site Hot Desk Facility. Further information: https://tilda.tcd.ie/data/accessing-data/ (accessed on 5 May 2021).

## Supplementary Materials

The following are available online at https://www.mdpi.com/article/10.3390/geriatrics6020050/s1, Figure S1: Sample for Analysis. AS; active stand, HAC: Health Assessment Centre.
