# Supplementary figures and images for: Eight Orthostatic Haemodynamic Patterns in The Irish Longitudinal Study on Ageing (TILDA): Stability and Clinical Associations after 4 Years
Source: Geriatrics (Basel). 2021 May 11;6(2):50. doi: 10.3390/geriatrics6020050 (PMC8162355; doi:10.3390/geriatrics6020050)

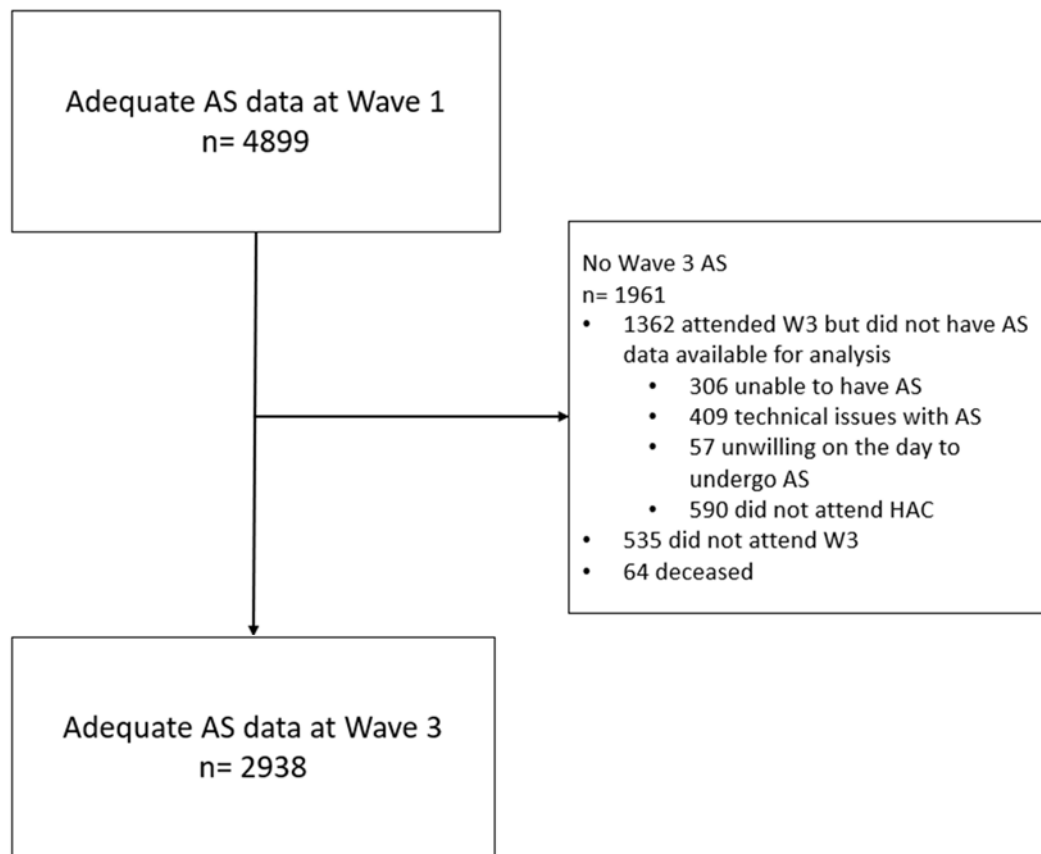

**Figure S1:** Sample for Analysis. AS; active stand, HAC: Health Assessment Centre

Supplement: Supplementary file 1 [file geriatrics-06-00050-s001.zip › geriatrics-1196029-supplementary.pdf]
